# The feasibility of establishing a hamster model for HBV infection: *in vitro* evidence

**DOI:** 10.1128/mbio.02615-24

**Published:** 2024-09-27

**Authors:** Hu Zhang, Yanan Liu, Cheng-Der Liu, Zhongde Wang, Haitao Guo

**Affiliations:** 1 Cancer Virology Program, UPMC Hillman Cancer Center, University of Pittsburgh, School of Medicine, Pittsburgh, Pennsylvania, USA; 2 Department of Microbiology and Molecular Genetics, University of Pittsburgh School of Medicine, Pittsburgh, Pennsylvania, USA; 3 Department of Animal, Dairy and Veterinary Sciences, Utah State University, Logan, Utah, USA; The University of North Carolina at Chapel Hill, Chapel Hill, North Carolina, USA

**Keywords:** hepatitis B virus, hamster hepatocytes, NTCP, cccDNA

## Abstract

Chronic hepatitis B virus (HBV) infection remains a significant public health burden with no cure currently available. The research to cure HBV has long been hampered by the lack of immunocompetent small animal models capable of supporting HBV infection. Here, we set out to explore the feasibility of the golden Syrian hamster as an immunocompetent small rodent model for HBV infection. We first started with *in vitro* assessments of the HBV replication cycle in primary hamster hepatocytes (PHaHs) by adenoviral HBV (Ad-HBV) transduction. Our results demonstrated that PHaHs support HBV reverse transcription and subsequent cccDNA formation via the intracellular recycling pathway. Next, with luciferase reporter assays, we confirmed that PHaHs support the activities of all HBV major promoters. Then, we transduced PHaHs with an adenoviral vector expressing HBV receptor human Na+/taurocholate cotransporting polypeptide NTCP (Ad-huNTCP), followed by HBV inoculation. While the untransduced PHaHs did not support HBV infection, Ad-huNTCP-transduced PHaHs supported *de novo* cccDNA formation, viral mRNA transcription, and expression of viral antigens. We then humanized the amino acid (aa) residues of hamster NTCP (haNTCP) critical for HBV entry, aa84-87 and aa157-165, and transfected HepG2 cells with constructs expressing wild-type haNTCP and humanized-haNTCP, H84R/P87N and H84R/P87N/G157K/M160V/M165L, respectively, followed by HBV inoculation. The results showed that the humanization of H84R/P87N alone was sufficient to support HBV infection at a level comparable to that supported by huNTCP. Taken together, the above *in vitro* evidence supports the future direction of humanizing haNTCP for HBV infection *in vivo*.

**IMPORTANCE:**

One of the biggest challenges in developing an HBV cure is the lack of immunocompetent animal models susceptible to HBV infection. Developing such models in mice has been unsuccessful due to the absence of a functional HBV receptor, human NTCP (huNTCP), and the defect in supporting viral cccDNA formation. In search of alternative models, we report herein multiple lines of *in vitro* evidence for developing a golden Syrian hamster model for HBV infection. We demonstrate that the primary hamster hepatocytes (PHaHs) support HBV replication, transcription, and cccDNA formation, and PHaHs are susceptible to *de novo* HBV infection in the presence of huNTCP. Furthermore, expressing hamster NTCP with two humanized residues critical for HBV entry renders HepG2 cells permissive to HBV infection. Thus, our work lays a solid foundation for establishing a gene-edited hamster model that expresses humanized NTCP for HBV infection *in vivo*.

## OBSERVATION

Chronic hepatitis B virus (HBV) infection affects more than 296 million people globally and approximately 820,000 people die each year from HBV-related complications such as cirrhosis and hepatocellular carcinoma (HCC). Although antiviral therapeutics for HBV are available, they rarely lead to a functional cure for chronic HBV infection, which is urgently needed (1). One of the major impediments in developing a cure for HBV is the lack of immunocompetent animal models permissive to HBV infection (2). HBV has an extremely narrow host tropism, determined both by host species-specific viral receptor, specifically the transmembrane Na^+^/taurocholate cotransporting peptide (NTCP) on the cell surface (3), and other host factors needed to complete the lifecycle of HBV after viral entry (4). To date, mouse models supporting HBV infection are limited to immunodeficient humanized mice transplanted with human hepatocytes (5). HBV transgenic mice do not support the formation of viral covalently closed circular DNA (cccDNA), the bona fide viral transcription template, via the intracellular cccDNA amplification mechanism (6); genetically modified mice carrying humanized NTCP support viral entry but fail to establish HBV infection due to the failure of *de novo* cccDNA formation in mouse hepatocytes (7). While the intracellular barrier for HBV infection in mouse hepatocytes remains poorly understood (8, 9), assessing the permissiveness of hepatocytes from other commonly used laboratory animal species to HBV infection represents an alternative approach toward the development of novel HBV animal models. Here, we report that the primary hamster hepatocytes (PHaHs) from golden Syrian hamster are permissive to HBV cccDNA formation and huNTCP-mediated viral infection and that humanized hamster NTCP could support HBV infection in HCC cell line HepG2, thus providing the feasibility for further developing a hamster model with humanized NTCP to support HBV infection *in vivo*.

**Fig 1:**
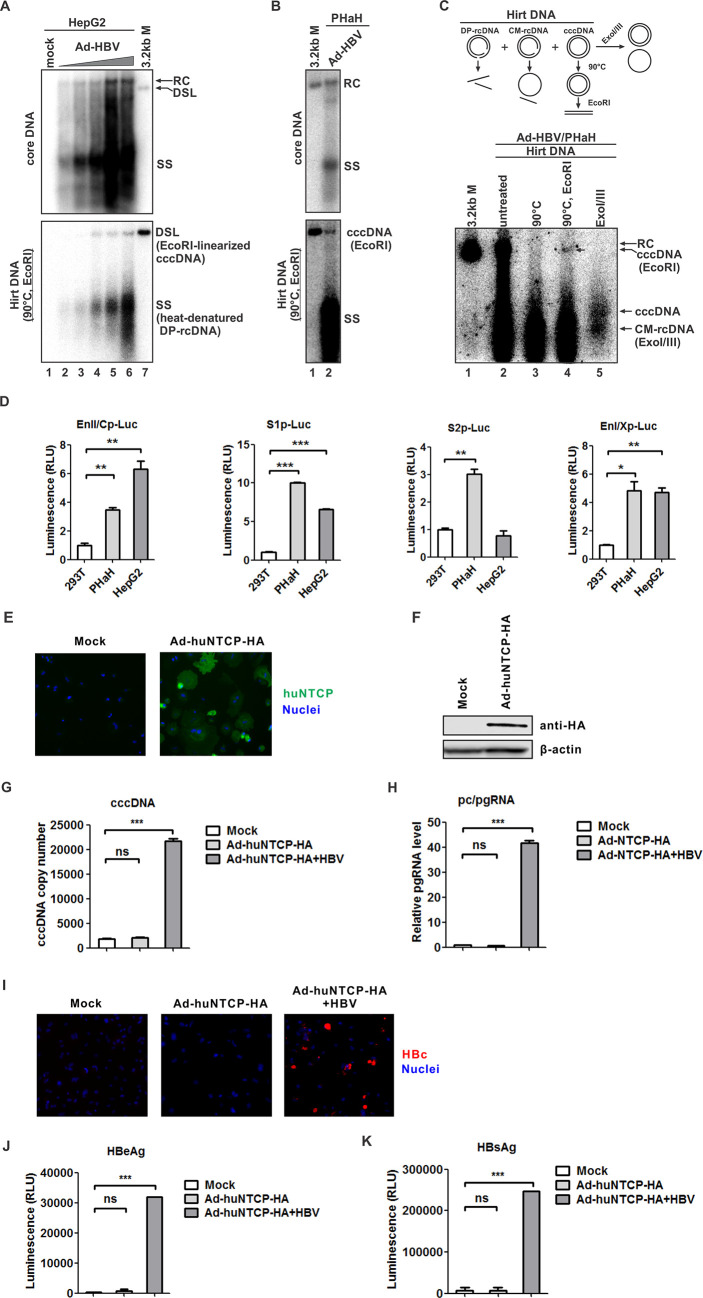
Primary hamster hepatocytes (PHaHs) support HBV cccDNA formation and huNTCP-mediated HBV infection. (**A**) HepG2 cells were either left untransduced (lane 1) or transduced by Ad-HBV at increasing MOIs of 20 (lane 2), 40 (lane 3), 80 (lane 4), 160 (lane 5), and 320 (lane 6) for 6 days. HBV cytoplasmic core DNA (DNA replicative intermediates) (top panel) and cccDNA (bottom panel) were detected by Southern blot. For cccDNA detection, Hirt DNA samples were heat denatured at 90°C and then digested by EcoRI, by which turned the deproteinated relaxed circular DNA (DP-rcDNA) into single-stranded (SS) DNA and cccDNA was linearized into double-stranded linear (DSL) form, a common practice to highlight cccDNA on Southern blot. The 3.2 kb unit-length linear HBV DNA served as size marker (lane 7). (**B**) PHaHs were transduced with Ad-HBV at MOI of 200 for 6 days. Intracellular HBV core DNA and Hirt DNA were extracted and subjected to Southern blot analysis. (**C**) The Hirt DNA sample from (**B**) was aliquoted and subjected to various treatments as schematically illustrated (top panel) and subsequent Southern blot assay (bottom panel), including untreated (lane 2), 90°C heat denaturation for 5 min (lane 3), 90°C heat denaturation followed by EcoR I linearization (lane 4), or directly digested by exonuclease I and III (ExoI/III) (lane 5). CM-rcDNA: closed minus-strand rcDNA. (**D**) Nonhepatic 293T cells, PHaHs, and hepatoma HepG2 cells were co-transfected by each indicated HBV promoter firefly luciferase (Luc) reporter plasmid and CMV promoter *Renilla* luciferase reporter plasmid pRL-CMV (20:1 ratio) for 3 days, followed by dual luciferase assay. The firefly luciferase signals were normalized by *Renilla* luciferase signals and plotted as fold change against that in 293T cells (mean ± SD, *n* = 3; *P < 0.05, **P < 0.01, ***P < 0.001). EnII/Cp: enhancer II and core promoter, S1p: S1 promoter; S2p: S2 promoter; EnI/Xp: enhancer I and X promoter. (**E, F**) PHaH cells were transduced with Ad-huNTCP-HA at MOI of 20 for 2 days. The expression of huNTCP-HA was analyzed by immunofluorescence (**E**) and Western blot (**F**). Cell nuclei were counter-stained by DAPI (**E**) and β-actin served as loading control (**F**). (**G–K**) PHaH cells were transduced with Ad-huNTCP-HA at MOI of 20 for 2 days, followed by HBV infection at MOI of 500 for 3 days. The intracellular cccDNA was quantified by qPCR and normalized by hamster mitochondrial DNA (mean ± SD, *n* = 3; ***P < 0.001) (**G**); HBV 3.5 kb pc/pgRNA was analyzed by RT-qPCR and normalized by hamster GAPDH mRNA, data are presented as fold change versus mock control (mean ± SD, *n* = 3; ***P < 0.001) (**H**); intracellular HBc was detected by immunofluorescence (**I**); and HBeAg (**J**) and HBsAg (**K**) in the supernatant were measured by CLIA (mean ± SD, *n* = 3; ***P < 0.001).

Due to the low transfection efficiency of PHaHs, we employed an Adenoviral vector to deliver genes of interest into PHaHs through transduction. In the positive control HepG2 cells, Ad-HBV transduction titer dependently resulted in HBV DNA replication and cccDNA formation as shown by Southern blot (Fig. 1A). Remarkably, cccDNA was readily detected in Ad-HBV-transduced PHaH cells (Fig. 1B). The cccDNA detected here is shown as a 3.2 kb band, which is derived from the heat-treated cccDNA followed by EcoR I linearization (Fig. 1C, lanes 1–4). Additionally, the authenticity of cccDNA produced in Ad-HBV-transduced PHaH cells was further validated by direct treatment by exonuclease ExoI/III, which left the undigested cccDNA and a minus-strand only circular DNA derived from ExoI/III-digested closed minus-strand rcDNA (CM-rDNA) being detected by Southern blot (Fig. 1C, lane 5). Both methods could effectively separate and highlight cccDNA from other protein-free HBV DNA species in the Hirt DNA extract (10, 11). All the above results clearly demonstrate that PHaHs support HBV cccDNA formation via the intracellular recycling pathway.

Then, we assessed whether PHaHs support HBV promoter activity using the luciferase reporter assay. HepG2 cells and the nonhepatic 293T cells served as positive and negative controls, respectively. As shown in Fig. 1D, PHaHs supported all four major HBV enhancers and promoters (EnII/Cp, S1p, S2p, and EnI/Xp) at comparable levels with that in HepG2 cells. It is worth noting that S2p activity was not strictly liver-specific as previously reported (12), but it was somewhat more active in PHaHs (Fig. 1D, third panel from the left). These data indicate that the PHaHs cells are equipped with all the essential hepatic transcription factors for HBV transcription.

Next, we set out to test HBV infection in PHaHs transduced with Ad-huNTCP-HA. To this end, we first used HepG2 cells to optimize the experimental conditions. To minimize the dedifferentiation of primary cells, we designed a 5-day transduction and infection scheme (Fig. S1A). Two-day Ad-huNTCP-HA transduction allowed a viral titer-dependent expression of huNTCP on the cell membrane (Fig. S1B and C), followed by HBV infection (MOI: 500) for an additional 3 days to produce detectable HBV parameters including HBc, cccDNA, and HBeAg (Fig. S1D through F). Notably, Ad-hNTCP-HA transduction at MOI 100 and 200 and subsequent HBV infection caused cytopathic effect (CPE) and reduced HBV infection (Fig. S1D through F). Therefore, Ad-huNTCP-HA transduction at MOI 20 to 50 appeared to be optimal for the downstream HBV infection, and, considering the fragility of primary cells, we performed Ad-huNTCP-HA transduction of PHaHs at MOI of 20. With a detectable level of huNTCP expression in PHaHs at 2-day post-transduction (Fig. 1E and F), the subsequent HBV infection yielded significant levels of HBV cccDNA and its transcription products pc/pgRNA (Fig. 1G and H). Additionally, viral antigens including the intracellular HBc and secreted HBeAg and HBsAg were also detected (Fig. 1I through K). While the primary human hepatocytes (PHuHs) were susceptible to HBV and the infection could be further enhanced upon Ad-huNTCP-HA transduction; in marked contrast, PHaHs without huNTCP expression could not support HBV infection (Fig. S2). Collectively, the data demonstrate that PHaHs are permissive to huNTCP-mediated HBV infection.

**Fig 2:**
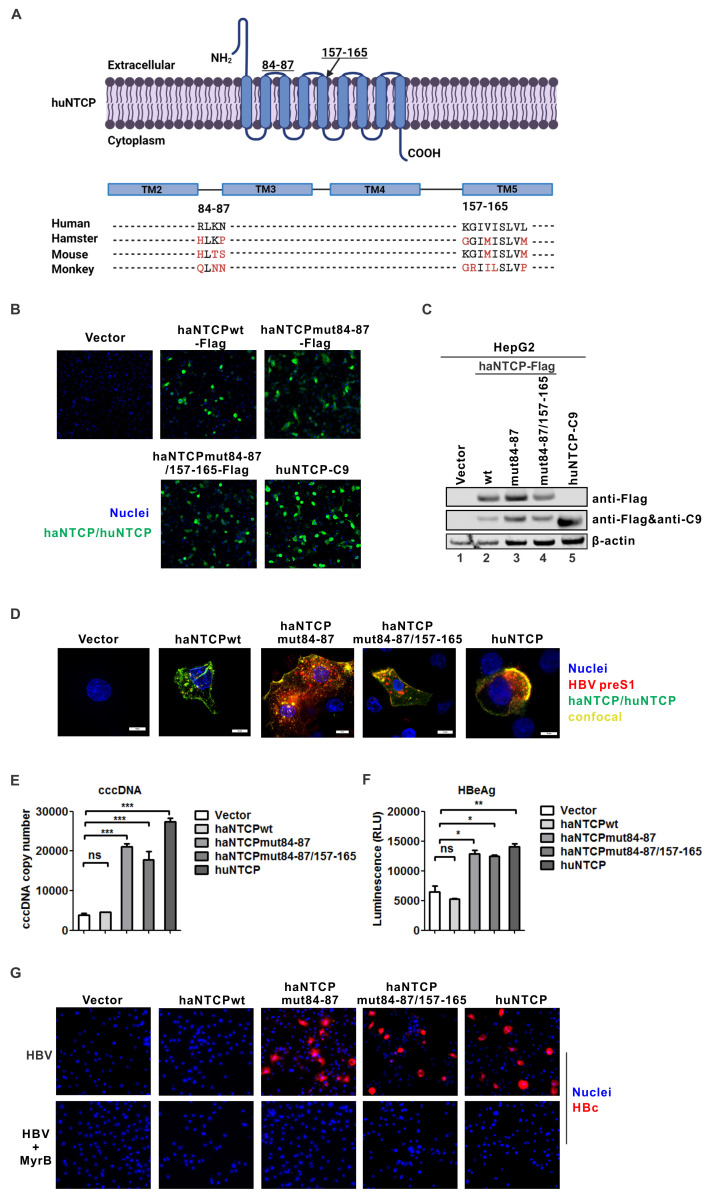
Humanized hamster NTCP (haNTCP) supports HBV infection *in vitro*. (**A**) Membrane topology of NTCP, a nine transmembrane (TM) protein (top panel). The sequence alignment highlights the aa 84–87 and 157–165 domains of NTCP protein among human (NP_003040.1), golden Syrian hamster (XP_005072871.1), mouse (NP_035517.1), and rhesus monkey (ALX38773.1) (bottom panel). The polymorphisms from hamster, mouse, and monkey NTCP in these two domains are indicated in red. Created with Biorender.com. (**B–D**) HepG2 cells were transfected with empty vector, wild-type haNTCP (haNTCPwt-Flag), humanized haNTCP (haNTCPmut84-87-Flag and haNTCPmut84-87/157-164-Flag), or human NTCP (huNTCP-C9) for 2 days; the cells were subjected to haNTCP-Flag and huNTCP-C9 immunofluorescence (**B**) and Western blot (**C**) using anti-Flag and anti-C9 antibodies, respectively. (**D**) Another set of transfected cells was probed with preS1-TAMRA, followed by haNTCP-Flag and huNTCP-C9 immunofluorescence for detection of preS1 and NTCP colocalization by confocal microscopy. (**E–G**) The above transfected HepG2 cells were infected with HBV (MOI: 500) for 3 days, the cells were collected for cccDNA qPCR and normalized by human mitochondrial DNA qPCR (mean ± SD, *n* = 3; ***P < 0.001) (**E**), and the supernatant samples were subjected to HBeAg CLIA (mean ± SD, *n* = 3; *P < 0.05, **P < 0.01) (**F**). (**G**) Another set of transfected HepG2 cells was left untreated or treated with HBV entry inhibitor MyrB (500 nM) during HBV infection (MOI: 500), and 3 days later, the cells were subjected to HBc immunofluorescence.

To establish a hamster model for HBV infection, one option is to make the transgenic hamster with a systemic or liver-specific expression of huNTCP; however, using a genome-editing approach to humanize the hamster NTCP (haNTCP) will be ideal, given that the critical sites on huNTCP for HBV binding/entry have been determined in previous mutagenesis and structural studies, including the first extracellular loop (aa 84–87) and N-terminal aa 157–165 of the fifth transmembrane domain (3, 7, 13–18) (Fig. 2A). Humanizing certain key residues in these two domains has rendered mouse and monkey NTCP susceptible to HBV entry in previous studies (17, 19–21). To assess whether humanized haNTCP could support HBV infection, we constructed plasmids expressing C-terminally Flag-tagged wild-type haNTCP (haNTCPwt-Flag) and humanized haNTCP with completely humanized aa 84–87 only (haNTCPmut84-87-Flag) or both aa 84–87 and 157–165 (haNTCPmut84-87/157-165-Flag). HepG2 cells were transfected with wt or humanized haNTCP expressing plasmids for 2 days, followed by HBV infection. Empty vector and plasmid expressing C-terminally C9-tagged huNTCP (huNTCP-C9) served as negative and positive control, respectively. All the NTCP proteins were successfully expressed at day 2 post-transfection (Fig. 2B and C). The preS1-TAMRA binding assay demonstrated that HepG2 cells expressing huNTCP and humanized haNTCPs, but not the mock- or wt haNTCP-transfected cells, were positively probed by preS1 peptide and colocalization signals between the probe and NTCPs were detected (Fig. 2D). In line with these, HBV inoculation led to cccDNA formation, HBeAg secretion, and intracellular HBc expression in HepG2 cells transduced with huNTCP, haNTCPmut84-87 and haNTCPmut84-87/157-165, but not the mock- or wt haNTCP-transfected cells (Fig. 2E and F and G (top panel)). All the detected HBV parameters were comparable among positive samples, though the signals were slightly higher in huNTCP-transfected cells, which might be due to a higher expression of NTCP protein (Fig. 2B and C). Notably, the haNTCPmut84-87 supported a similar level of HBV infection as haNTCPmut84-87/157-165 did (Fig. 2E through G), suggesting that humanizing aa 84–87 of haNTCP is sufficient to support HBV entry, which is consistent with the mouse NTCP humanization studies (7, 15). It is also worth noting that one critical residue G158 for HBV binding is conserved in human, hamster, and mouse NTCPs (17, 18) (Fig. 2A). Furthermore, the infections were successfully blocked by HBV entry inhibitor Myrcludex B (MyrB) (Fig. 2G), further confirming the huNTCP- and humanized haNTCP-mediated HBV infection.

In summary, our study provides solid *in vitro* evidence that hamster hepatocytes support HBV cccDNA formation and that humanizing hamster NTCP can breach the species barrier for HBV infection. These findings justify the future efforts to humanize hamster NTCP *in vivo* through gene editing. An immunocompetent and infection-competent hamster model would address the critical gaps in developing a functional cure for HBV.

## Data Availability

All data are included in the paper or available upon request.

## SUPPLEMENTAL MATERIAL

Supplemental material mbio.02615-24-s0001.pdf Additional experimental details and Figures S1 and S2.

ASM does not own the copyrights to Supplemental Material that may be linked to, or accessed through, an article. The authors have granted ASM a non-exclusive, world-wide license to publish the Supplemental Material files. Please contact the corresponding author directly for reuse.
