# Supplementary material for: The feasibility of establishing a hamster model for HBV infection: in vitro evidence
Source: mBio. 2024 Sep 27;15(11):e02615-24. doi: 10.1128/mbio.02615-24 (PMC11559161; doi:10.1128/mbio.02615-24)
Supplement: Supplemental material — Additional experimental details and Figures S1 and S2. [file mbio.02615-24-s0001.pdf]

# SUPPLEMENTAL MATERIALS

## SUPPLEMENTAL MATERIAL AND METHODS

**Cell lines and viruses.** HepG2, 293T and Ad293 cells were purchased from ATCC and cultured in DMEM/F12 medium (Cytiva, hyclone, Cat# SH30023.01) supplemented with 10% fetal bovine serum (R&D Systems, Cat# S10350), 100 U/ml penicillin and 100 μg/ml streptomycin (Corning, Cat# 30-002-CI). The tetracycline (tet)-inducible HBV stable cell line HepAD38 cells were cultured in the same condition as HepG2 cells with additional 1 μg/ml tet (Gibco, Cat# A39246). HBV infectious particles were collected and concentrated from the supernatant of induced HepAD38 cells (tet-) according to our previously published protocol (1). The recombinant Adenoviral HBV (Ad-HBV) carrying HBV pregenomic RNA (pgRNA) coding sequence under the control of CMV-IE promoter has been described previously (2). The Ad-HBV stock was expanded by transducing Ad293 cells for 2–3 days until the cells exhibited significant cytopathic effects (CPE). The supernatant was then discarded, and the cells were collected by centrifugation at 1,000 ×g. After washing with 1× DPBS (Corning, Cat# 21-031-CV), the cell pellet was suspended in 10 mM Tris-HCl, pH 8.1, and subjected to three cycles of freezing and thawing to obtain the supernatant. Recombinant adenoviral vector Ad-huNTCP-HA expression C-terminally HA-tagged human NTCP was purchased from ABM (Cat# 131659A) and expanded with the same methods as Ad-HBV. The recombinant Adenoviruses were purified by Adeno-X Maxi Purification Kit (Takara Bio, Cat# 631533) and titrated by Adeno-X qPCR Titration Kit (Takara Bio, Cat# 632252). The virus stocks were aliquoted and stored in -80 °C.

**Plasmids and transfection.** Plasmid pcDNA6-NTCP expressing the C-terminally rhodopsin C9-tagged human NTCP (huNTCP-C9) was provided by Dr. Wenhui Li (National Institute of Biological Sciences, Beijing, China) (3). To construct plasmids to express the C-terminally Flag-

tagged wild type golden Syrian hamster NTCP (haNTCPwt-Flag) and humanized haNTCP containing H84R/P87N mutations (haNTCPmut84-87-Flag) and H84R/P87N/G157K/M160V/M165L mutations (haNTCPmut84-87/157-165-Flag), the corresponding DNA sequences of wt (Genbank Accession Number: XM_005072814) and mutant haNTCP ORF fused with a 3' Flag tag coding sequence were chemically synthesized by Genscript and cloned into the expression ready pcDNA3 vector. The HBV promoter firefly luciferase report plasmids, including EnII/Cp-Luc, S1p-Luc, and S2p-Luc, have been described previously (4). To construct the HBV enhancer I and X promoter luciferase reporter plasmid EnI/Xp-Luc, a DNA fragment containing HBV EnI/Xp DNA sequence (nt 950-1375) was inserted into the Kpn I and Hind III restriction sites of the pGL3-Basic vector (Promega, Cat# E1751). The CMV promoter *Renilla* luciferase reporter plasmid pRL-CMV was purchased from Promega (Cat# E2261). Plasmid transfection was conducted with Lipofectamine 3000 (ThermoFisher, Cat# L3000150) according to manufacturer's manual.

**Primary hamster hepatocytes (PHaHs) isolation and culturing.** The method for isolating primary hepatocytes from golden Syrian hamster was adopted as described previously (5). Liver of hamster was perfused with HBSS perfusion buffer (Gibco, #14175095) containing HEPES and EDTA and then digested with HBSS digestion buffer (Gibco, #24020117) containing Liberase (Roche, #5401119001). The digested liver was ruptured with forceps and gently released with cell lifter. The suspended cells were filtered by 70 μm cell strainer and purified with Percoll solution (Santa Cruz, Cat# sc-500790) for high viability. The hepatocytes were seeded on collagen (Corning, Cat# 354236, 1/100 dilution) coated plates in EMEM (Sigma, Cat# M0894) medium supplied with 2 mg/mL albumin (Sigma, Cat# A2153), 2 mg/mL Galactose (Sigma, Cat# G5388), 0.1 mg/mL Omithine (Sigma, Cat# O2375), 30 μg/mL Proline (Sigma, Cat# P4655), 2.38 mg/mL

HEPES (Sigma, Cat# H3375), 0.05 µg/mL $ZnCl_2$ (Sigma, Cat# Z0152), 0.02 µg/mL $CuSO_4$ (Sigma, Cat# C7631), 0.08 µg/mL (Sigma, Cat# Z0251), 1.25 µg/mL $MnSO_4$ (Sigma, Cat# M7899), 0.15 mg/mL Glutamine (Sigma, Cat# G3126), 0.1 µM Dexamethasone (Sigma, Cat# D4902), 1× ITS (Corning 1000×, Cat# 354350), 20 ng/mL hEGF (Sigma, Cat# 62253-63-8), 2.2 mg/mL $NaHCO_3$, and 100 U/mL penicillin and 100 µg/mL streptomycin. The cells were cultured in 37°C, 5% $CO_2$ incubator. The animal experiments were performed under protocol approved by the Institutional Animal Care and Use Committee at the Utah State University (protocol number 13758).

**Primary human hepatocytes (PHuH).** Freshly isolated PHuH cells were obtained through the Human Liver Tissue and Hepatocyte Research Resource (HLTHRR) at University of Pittsburgh (funded by NIDDK project# 1R24DK139775-01) and cultured as previously described (6).

**Adenovirus transduction and HBV infection.** To perform adenoviral transduction of HepG2 and PHaH cells, the recombinant adenovirus was added to the medium of cell monolayers at indicated multiplicity of infection (MOI) (aka viral genome equivalent per cell (vge/cell). After incubation for 24 h, the medium was replaced, and the cells were cultured for indicated time of duration. HBV infection was performed as described previously (7) with minor modifications. Briefly, cells were seeded in the collagen-coated 6-well plate with density of $1.2\times10^6$ cells/well and cultured in regular DMEM/F12 medium for overnight, and then the culture medium was switched to Cellartis Hepatocyte Maintenance Medium (HMM) (Takara Bio, Cat# Y30051) for overnight. Then cells were incubated with HBV inoculum at MOI of 500 that was diluted in HMM containing 4% PEG-8000. One day post infection, the HBV inoculum was removed, and the cells were incubated in HMM for another day. In the following days, the cells were maintained in primary hepatocytes

maintenance medium (PMM) before harvest. PMM was prepared according to a published recipe (3).

**Nucleic acids analyses.** HBV cytoplasmic core DNA and whole cell Hirt DNA were extracted and analyzed by Southern blot as previously described (8, 9). For HBV CM-rcDNA detection, the Hirt DNA samples were digested with ExoI/III (7 Unit ExoI and 35 Unit ExoIII per reaction) and then subjected to Southern blot analysis according to publications (6, 10). An aliquot of Hirt DNA sample was saved for human or golden Syrian hamster mitochondrial DNA (mtDNA) qPCR for normalization purpose. The human mtDNA qPCR primers are 5'-CGGGCACAGTGATTAT-3' (forward) and 5'-ATGTAGCCGTTGAGTTG-3' (reverse). The hamster mtDNA qPCR primers are 5'-AGTAGCACATATTTGTCGAGA-3' (forward) and 5'-CCGACATGAAGAAATAAGCAA-3' (reverse). HBV cccDNA qPCR were performed according to literature (6, 11). Briefly, to reduce contamination of HBV rcDNA in the qPCR detection of cccDNA, the Hirt DNA samples was first heated at 85°C for 5 min to denature rcDNA into single-stranded DNA, followed by Plasmid-safe ATP-dependent DNase (PSAD) (LGC Biosearch Technologies, Cat# E3101K) treatment at 37º C for 16 h. The PSAD reaction was then stopped by heat inactivation at 70º C for 30 min, and then the samples were further purified by DNA clean-up spin column (Zymo Research, Cat# 11-303). Real-time PCR amplification of 3 µl cleaned cccDNA sample (1/10 from eluted cccDNA) was performed in a 20 µl reaction containing 1.0 µM forward primer (5'- GTCTGTGCCTTCTCATCTGC -3'), 1.0 µM reverse primer (5'-AGTAACTCCACAGTAGCTCCAAATT -3'), and 0.2 µM TaqMan probe (5'-FAM-TTCAAGCCTCCAAGCTGTGCCTTGGGTGGC-TAMRA-3'). Total RNA samples were extracted from the whole cells by Tri Reagent (Millipore Sigma, Cat# T9424) and treated with RNase-free DNase I (Promega, Cat# M6101) for 30 min, followed by DNase I inactivation at 70

ºC for 10 min. The DNase I-treated RNA samples were subjected to Reverse Transcription (RT) using Superscript IV VILO Master Mix (ThermoFisher, Cat# 11756050). The RT products were subjected to qPCR using different primer pairs. For HBV 3.5 kb mRNA (precore mRNA and pgRNA (pc/pgRNA), forward primer is 5'-GGTCCCCTAGAAGAAGAACTCCCT-3'; Reverse primer is 5'-CATTGAGGTTCCCGAGATTGAGAT-3'. For human GAPDH, the forward primer is: 5'-TCTGACTTCAACAGCGACACC-3'; the reverse primer is 5'-CTGTTGCTGTAGCCAAATTCGTT-3'. For hamster GAPDH, forward primer is 5'-AGATTGCTGCCATCAATGACC-3', reverse primer is 5'-ATTCTCAGCCTTGACTGTGC-3'. The FastStart Essential DNA Probes Master (Roche) and FastStart Universal SYBR Green Master (Roche) were used to assemble TaqMan and SYBR Green qPCR reactions, respectively. The qPCR was run by Roche LightCycler 96 under the following thermal cycling conditions: 10 min at 95˚C, followed by 15 sec at 95˚C and 1 min at 61˚C for 50 cycles. The cccDNA qPCR data was normalized by the cellular mitochondrial DNA quantitation. Graphpad Prism 10 software was used to generate the histogram and calculate the t-test statistic data.

**Western blot, ELISA, CLIA, and luciferase assay.** To perform Western blot, the cells were lysed in 1% NP40 lysis buffer containing 1× protease inhibitor cocktail (Cell Signaling, Cat# 5871), followed by sonication at 33% amplitude, 10 sec impulse and 2 sec rest for 6 times on ice using Epishear Probe Sonicator (Active Motif). The sonicated samples were centrifuged at 10,000 ×g for 10 min. The supernatant was used as the Western blot samples. The prepared samples were mixed with 2× Laemmli buffer and loaded on a Novex WedgeWell 4-12% Tris-Glycine Gel (Invitrogen, Cat# XP04122) for electrophoresis, and then proteins were transferred onto an Immobilon-FL polyvinylidene difluoride (PVDF) membrane (Millipore, Cat# IPFL00010). The membrane was then blocked with WesternBreeze blocking buffer (Invitrogen, Cat# WB7050) and

probed with specific antibodies. The antibodies used for probing the target proteins were mouse anti-HA (Genscript, Cat# A01244, 1 mg/ml), mouse anti-Flag (Millipore Sigma, Cat# F3165), mouse anti-C9 (Santa Cruz, rhodopsin Antibody (1D4), and mouse anti-β-actin (Santa Cruz, Cat# sc-47778) at dilution of 1:1000. The secondary antibodies include WesternSure Goat anti-Mouse HRP Secondary Antibody (LI-COR, Cat# 926-80010), IRDye 680RD Goat anti-Mouse IgG Secondary Antibody (LI-COR, Cat# 926-68070). The Western blot chemiluminescence or fluorescence signals were scanned by LI-COR Odyssey Fc. The supernatant samples were subjected to HBeAg ELISA (CUSABIO, Cat# CSB-E13557h) or chemiluminescent immunoassay (CLIA) (Autobio, Cat# CL0312-2) and HBsAg CLIA (Autobio, Cat# CL0310-2) according to the manufacturers' manuals. Luciferase activities in cell cultures were analyzed using a dual luciferase assay kit (Promega, Cat# E1980). ELISA, CLIA, and Luciferase signals were measured by the BioTek Synergy 2 Multi-Mode Reader.

**Mycludex B and preS1-probe.** Myrcludex B (MyrB), a synthetic N-acylated HBV preS1 peptide (amino acids (aa) 2-48) that can bind to huNTCP and block HBV entry (12), was kindly provided by Dr. Stephan Urban (University of Heidelberg, Heidelberg, Germany). The C-terminal tetramethylrhodamine-labeled, N-terminally myristoylated HBV preS1 peptide (aa 2-48, preS1-probe or preS1-TAMRA) was a gift from Dr. Koichi Watashi (National Institute of Infectious Diseases, Tokyo, Japan) (13).

**Immunofluorescence and confocal.** The supernatant of cells in a 96-well plate was discarded and the cells were fixed with 4% paraformaldehyde for 20 min and permeabilized in 1× PBS solution containing 0.5% Triton X-100 for 1 h at room temperature. Subsequently, cells were blocked with IFA blocking buffer (10% FBS plus 2% bovine serum albumin in 1× PBS) for 1 h at room temperature, and then incubated with rabbit anti-HBcAg (Dako, cat# B0586), mouse anti-HA

(Genscript, Cat# A01244), anti-Flag (Millipore Sigma, Cat# F3165), or anti-C9 (Santa Cruz, Cat# sc-57432) at dilution of 1:250 for overnight. After washing by 1× PBS, cells were incubated with goat anti-mouse IgG (H+L) Cross-Adsorbed Secondary Antibody, Alexa Fluo 488 (Thermo Fisher Scientific, Cat# A11001,) or goat anti-rabbit IgG (H+L) Cross-Adsorbed Secondary Antibody, Alexa Fluo 594 (Thermo Fisher Scientific, Cat# A11012), and cell nuclei were counterstained with DAPI (4',6-diamidino-2-phenylindole, ThermoFisher, Cat# 62248) for 30 min at room temperature. Both primary and secondary antibodies were diluted in IFA blocking buffer. The cells were washed with 1×PBS and subjected to EVOS M5000 (Invitrogen) microscopy analysis under 20× objective lens. Images were analyzed using ImageJ software.

To perform preS1-TAMRA binding and colocalization assay with NTCP proteins, the following procedures were implemented. A glass coverslip was attached with a reinforcement label (Avery, Cat# 5720) and placed under UV light for 10 min for sterilization. The hole in the reinforcement label was used for cell attachment and grow. The hole on the coverslip was pretreated with a mixture of 20 μg/ml collagen (Corning, Cat# 354236) and 50 μg/ml Poly-D-Lysine (ThermoFisher, Cat# A3890401) for 1 h, followed by PBS washing for 3 times. When the coverslip surface became dry, it was ready for cell seeding. The transfected cells were treated with 100 nM of preS1-TAMRA probe at 37°C for 1 h. The cells were then trypsinized and seeded in the reinforcement hole on the coverslip and grew overnight to reach a sufficient stretching. Then the coverslip with cells was subjected to immunofluorescence steps as described above. After the antibody incubation steps were completed, the coverslip with cells were sealed up on a glass slide with enamel around the edge of the coverslip, followed by confocal detection under LDI-7 Laser Diode Illuminator - 89 North. The images were analyzed using ImageJ software.

**Statistical Analysis.** Data were analyzed using Prism 10 (GraphPad Software) with two-tailed unpaired t-tests. Data are expressed as mean ± standard deviation (SD), and p values of $<0.05$ are considered statistically significant.

## REFERENCES


1. Yan R, Zhang Y, Cai D, Liu Y, Cuconati A, Guo H. 2015. Spinoculation Enhances HBV Infection in NTCP-Reconstituted Hepatocytes. PLoS One 10:e0129889.
2. Guo JT, Zhou T, Guo H, Block TM. 2007. Alpha interferon-induced antiviral response noncytolytically reduces replication defective adenovirus DNA in MDBK cells. Antiviral Res 76:232-40.
3. Yan H, Zhong G, Xu G, He W, Jing Z, Gao Z, Huang Y, Qi Y, Peng B, Wang H, Fu L, Song M, Chen P, Gao W, Ren B, Sun Y, Cai T, Feng X, Sui J, Li W. 2012. Sodium taurocholate cotransporting polypeptide is a functional receptor for human hepatitis B and D virus. Elife 1:e00049.
4. Mao R, Nie H, Cai D, Zhang J, Liu H, Yan R, Cuconati A, Block TM, Guo JT, Guo H. 2013. Inhibition of hepatitis B virus replication by the host zinc finger antiviral protein. PLoS Pathog 9:e1003494.
5. Charni-Natan M, Goldstein I. 2020. Protocol for Primary Mouse Hepatocyte Isolation. STAR Protoc 1:100086.
6. Marchetti AL, Zhang H, Kim ES, Yu X, Jang S, Wang M, Guo H. 2022. Proteomic Analysis of Nuclear Hepatitis B Virus Relaxed Circular DNA-Associated Proteins Identifies UV-Damaged DNA Binding Protein as a Host Factor Involved in Covalently Closed Circular DNA Formation. J Virol 96:e0136021.
7. Mitra B, Wang J, Kim ES, Mao R, Dong M, Liu Y, Zhang J, Guo H. 2019. Hepatitis B Virus Precore Protein p22 Inhibits Alpha Interferon Signaling by Blocking STAT Nuclear Translocation. J Virol 93.
8. Guo H, Jiang D, Zhou T, Cuconati A, Block TM, Guo JT. 2007. Characterization of the intracellular deproteinized relaxed circular DNA of hepatitis B virus: an intermediate of covalently closed circular DNA formation. J Virol 81:12472-84.
9. Cai D, Nie H, Yan R, Guo J-T, Block TM, Guo H. 2013. A southern blot assay for detection of hepatitis B virus covalently closed circular DNA from cell cultures. Methods in molecular biology (Clifton, NJ) 1030:151-161.
10. Luo J, Cui X, Gao L, Hu J. 2017. Identification of an Intermediate in Hepatitis B Virus Covalently Closed Circular (CCC) DNA Formation and Sensitive and Selective CCC DNA Detection. J Virol 91.
11. Kim ES, Zhou J, Zhang H, Marchetti A, van de Klundert M, Cai D, Yu X, Mitra B, Liu Y, Wang M, Protzer U, Guo H. 2022. Hepatitis B virus X protein counteracts high mobility group box 1 protein-mediated epigenetic silencing of covalently closed circular DNA. PLoS Pathog 18:e1010576.
12. Ni Y, Lempp FA, Mehrle S, Nkongolo S, Kaufman C, Falth M, Stindt J, Koniger C, Nassal M, Kubitz R, Sultmann H, Urban S. 2014. Hepatitis B and D viruses exploit sodium taurocholate co-transporting polypeptide for species-specific entry into hepatocytes. Gastroenterology 146:1070-83.
13. Iwamoto M, Saso W, Sugiyama R, Ishii K, Ohki M, Nagamori S, Suzuki R, Aizaki H, Ryo A, Yun JH, Park SY, Ohtani N, Muramatsu M, Iwami S, Tanaka Y, Sureau C, Wakita T, Watashi K. 2019. Epidermal growth factor receptor is a host-entry cofactor triggering hepatitis B virus internalization. Proc Natl Acad Sci U S A 116:8487-8492.

## SUPPLEMENTAL FIGURE LEGENDS

**Fig. S1. Optimization of experimental conditions for Ad-huNTCP-HA transduction and subsequent HBV infection using HepG2 cells.** (A) Schematic diagram of experimental design. HepG2 cells were transduced with Ad-huNTCP-HA for 2 days at various MOIs and then infected with HBV at MOI of 500 for 3 days. (B, C) 2-day post Ad-huNTCP-HA transduction, the cells were collected for huNTCP-HA Western blot (B) and immunofluorescence (C) assays using anti-HA antibody. (D-F) 3-day post HBV infection, the cells were subjected to HBc immunofluorescence (D), cccDNA extraction and qPCR with normalization to human mitochondrial DNA (E), and the supernatant samples were collected for HBeAg ELISA assay (F). Cytopathic effect (CPE) was observed under HBV infection of the cells received Ad-huNTCP-HA at MOI of 100 and 200.

**Fig. S2. PHaHs are non-susceptible to HBV infection.** PHuHs and PHaHs were mock transduced or transduced with Ad-huNTCP-HA at MOI of 20 for 2 days, followed by HBV infection at MOI of 500 for additional 3 days. 2-day post Ad-huNTCP-HA transduction, the cells were collected for huNTCP-HA immunofluorescence (A) and Western blot (B) analyses using anti-HA antibody. 3-day post HBV infection, the cells were subjected to HBc immunofluorescence (C), and the supernatant was collected for HBeAg CLIA assay (mean ± SD, n=3; ** $p<0.01$, *** $p<0.001$) (D).

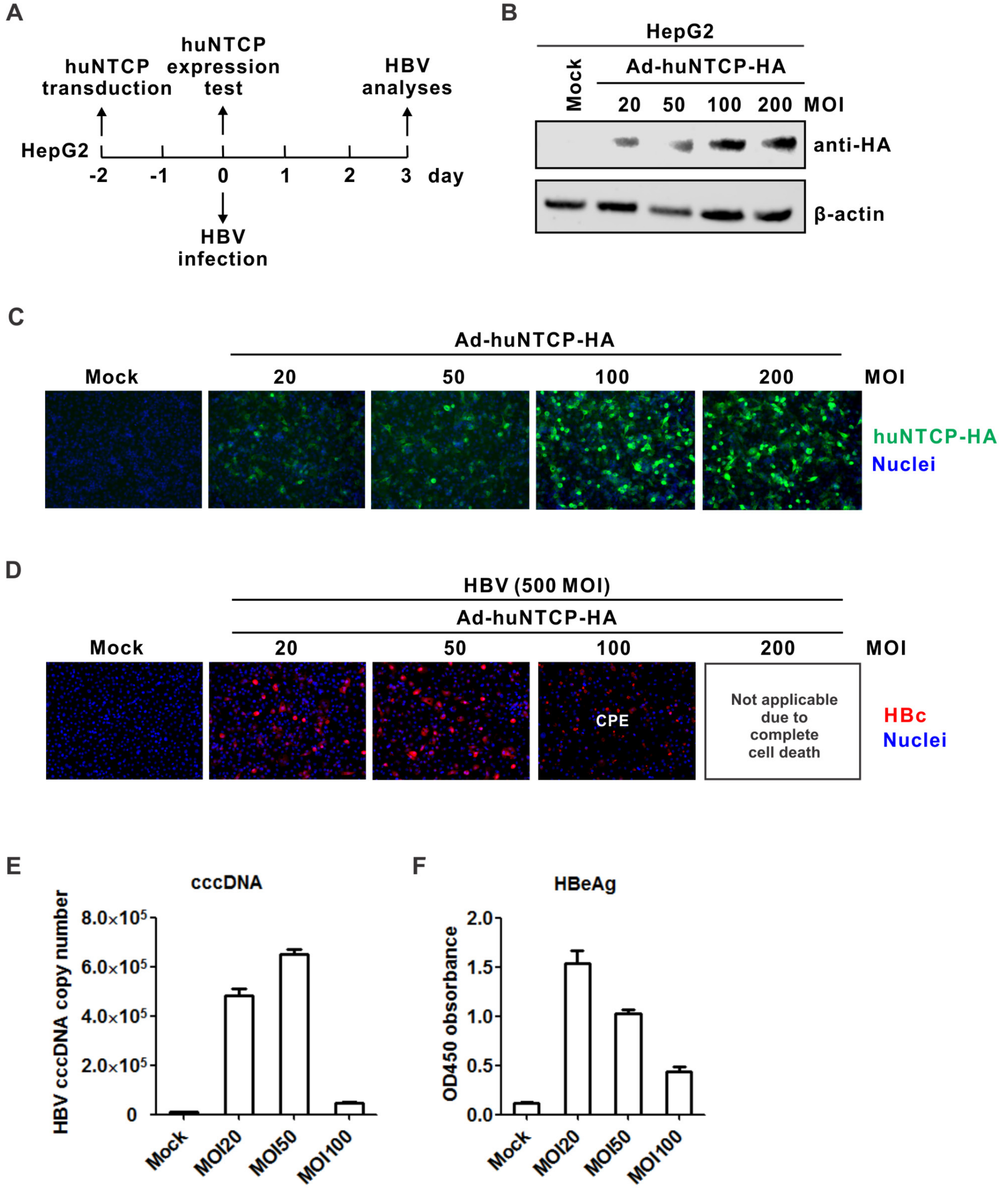
A
huNTCP transduction
huNTCP expression test
HBV analyses
HepG2
-2 -1 0 1 2 3 day
HBV infection
B
HepG2
Mock
Ad-huNTCP-HA
20 50 100 200 MOI
anti-HA
β-actin
C
Ad-huNTCP-HA
Mock 20 50 100 200 MOI
huNTCP-HA
Nuclei
D
HBV (500 MOI)
Ad-huNTCP-HA
Mock 20 50 100 200 MOI
CPE
Not applicable due to complete cell death
HBc
Nuclei
E
cccDNA
HBV cccDNA copy number
8.0×10⁵
6.0×10⁵
4.0×10⁵
2.0×10⁵
0
Mock MOI20 MOI50 MOI100
F
HBeAg
OD450 absorbance
2.0
1.5
1.0
0.5
0.0
Mock MOI20 MOI50 MOI100

**Figure S1**

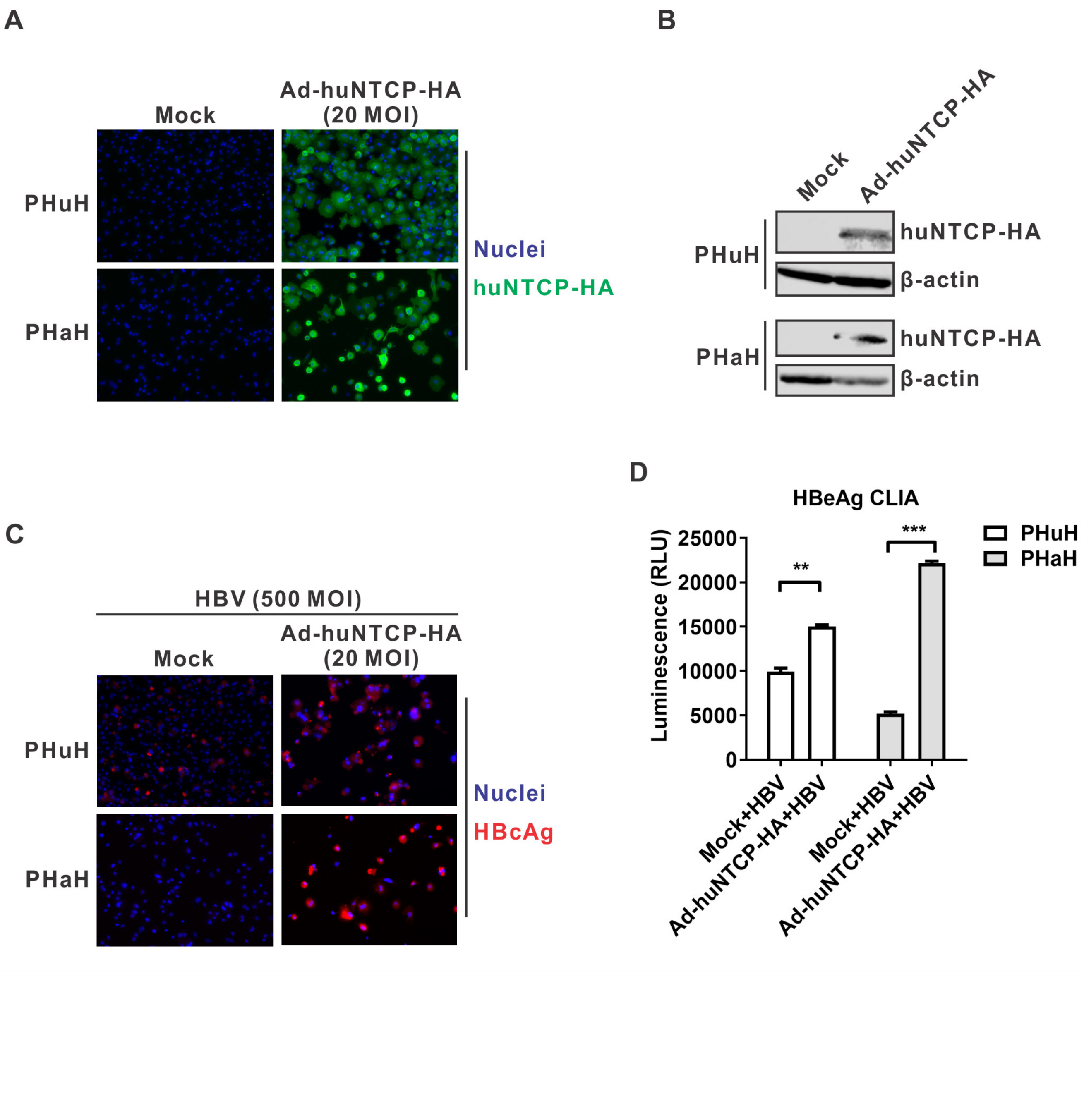
A
Mock
Ad-huNTCP-HA (20 MOI)
PHuH
PHaH
Nuclei
huNTCP-HA
B
Mock
Ad-huNTCP-HA
PHuH
huNTCP-HA
β-actin
PHaH
huNTCP-HA
β-actin
C
HBV (500 MOI)
Mock
Ad-huNTCP-HA (20 MOI)
PHuH
PHaH
Nuclei
HBcAg
D
HBeAg CLIA
Luminescence (RLU)
25000
20000
15000
10000
5000
0
**
***
PHuH
PHaH
Mock+HBV
Ad-huNTCP-HA+HBV
Mock+HBV
Ad-huNTCP-HA+HBV

**Figure S2**
